# *SLC30A3* as a Zinc Transporter-Related Biomarker and Potential Therapeutic Target in Alzheimer’s Disease

**DOI:** 10.3390/genes16111380

**Published:** 2025-11-13

**Authors:** Ruyu Bai, Zhiyun Cheng, Yong Diao

**Affiliations:** School of Medicine, Huaqiao University, Quanzhou 362021, China; bairuyu@hqu.edu.cn (R.B.); chengzhiyun@hqu.edu.cn (Z.C.)

**Keywords:** Alzheimer’s disease, zinc metabolism-related genes, *SLC30A3*, biomarker, therapeutic target

## Abstract

Background: Alzheimer’s disease (AD) is a progressive neurodegenerative disorder with unclear pathogenic mechanisms. Dysregulated zinc metabolism contributes to AD pathology. This study aimed to identify zinc metabolism-related hub genes to provide potential biomarkers and therapeutic targets for AD. Methods: We performed an integrative analysis of multiple transcriptomic datasets from AD patients and normal controls. Differentially expressed genes and weighted gene co-expression network analysis (WGCNA) were combined to identify hub genes. We then conducted Gene Set Enrichment Analysis (GSEA), immune cell infiltration analysis (CIBERSORT), and receiver operating characteristic (ROC) curve analysis to assess the hub gene’s biological function, immune context, and diagnostic performance. Drug-gene interactions were predicted using the DrugBank database. Results: We identified a single key zinc transporter–related hub gene, *SLC30A3*, which was significantly downregulated in AD and demonstrated potential diagnostic value (AUC 0.70–0.80). Lower *SLC30A3* expression was strongly associated with impaired synaptic plasticity (long-term potentiation, long-term depression, calcium signaling pathway, and axon guidance), mitochondrial dysfunction (the citrate cycle and oxidative phosphorylation), and pathways common to major neurodegenerative diseases (Parkinson’s disease, AD, Huntington’s disease, and amyotrophic lateral sclerosis). Furthermore, *SLC30A3* expression correlated with specific immune infiltrates, particularly the microglia-related chemokine *CX3CL1*. Zinc chloride and zinc sulfate were identified as potential pharmacological modulators. Conclusions: Our study systematically identifies *SLC30A3* as a novel biomarker in AD, linking zinc dyshomeostasis to synaptic failure, metabolic impairment, and neuroimmune dysregulation. These findings offer a new basis for developing targeted diagnostic and therapeutic strategies for AD.

## 1. Introduction

As the predominant type of dementia worldwide, Alzheimer’s disease (AD) is an irreversible neurodegenerative condition. AD is characterized by progressive cognitive decline and two core pathologies: extracellular amyloid-β (Aβ) plaques and intracellular neurofibrillary tangles (NFTs) [1,2]. These core pathologies are accompanied by a cascade of other detrimental processes, including chronic neuroinflammation, heightened oxidative stress, and widespread synaptic dysfunction [3,4]. Although significant progress has been made in understanding these pathological processes, the precise etiology of AD remains elusive. The existing pharmacological treatments for AD, which primarily consist of acetylcholinesterase inhibitors and the *N*-methyl-d-aspartate (NMDA) receptor antagonist memantine, offer only limited symptomatic relief and do not alter the fundamental progression of the disease [5]. The development of novel biomarkers to enable early diagnosis, alongside new therapeutic strategies for timely intervention, remains a critical priority. This urgency is driven by the current lack of any therapies capable of modifying the underlying course of the disease [6].

Growing evidence implicates metabolic dysregulation, including alterations in lipid, cholesterol, and metal ion homeostasis, as critical contributors to AD pathogenesis [7,8,9,10]. Among these, zinc metabolism has attracted increasing attention due to its pivotal role in synaptic transmission, regulation of Aβ aggregation, and modulation of neuroinflammatory responses [11,12,13]. Zinc dyshomeostasis has been documented both centrally and peripherally in AD patients. For example, recent neuroimaging studies have shown that reduced serum zinc levels are significantly correlated with elevated cerebral Aβ burden [14]. Zinc ions can directly bind tau protein, thereby accelerating its aggregation and exacerbating neurotoxicity [15]. Experimental studies have demonstrated that excessive synaptic zinc exposure can activate tau-related pathological signaling via the mTOR/p70S6K pathway, leading to site-specific hyperphosphorylation (e.g., at Ser356) and increased oxidative stress; conversely, pharmacological inhibition of this pathway has been shown to attenuate such zinc-induced effects [16]. In addition, zinc imbalance can potentiate Aβ-induced neurotoxicity and impair synaptic plasticity [17]. Emerging clinical and experimental evidence also suggests a connection between systemic zinc homeostasis and peripheral immune activation. For instance, in keratinocyte models, zinc deficiency has been associated with elevated CXCL10 expression via a PPARα-dependent mechanism, and zinc supplementation partially reversed this effect; these findings may have implications for neuroinflammatory processes in AD, although direct clinical confirmation in AD cohorts remains limited [18]. Collectively, these findings highlight zinc dyshomeostasis as a multifaceted driver of AD pathology, involving Aβ deposition, tau hyperphosphorylation, oxidative stress, and neuroimmune modulation [11,15,17,19]. However, the precise molecular mediators linking zinc metabolism to AD progression remain insufficiently characterized. Zinc homeostasis is regulated by a diverse group of proteins. We distinguish between the broad category of “zinc-binding proteins” (e.g., enzymes, transcription factors) and a specialized subset, the “zinc transporters” (e.g., ZIP/SLC39 families). The latter are membrane proteins that control zinc flux across membranes to maintain cellular concentrations.

In this study, we harnessed publicly available transcriptomic datasets and integrated bioinformatics analyses to identify zinc metabolism–related hub genes associated with AD. Differential expression and WGCNA were performed on the GSE48350 dataset, followed by GSEA to explore potential biological pathways. A diagnostic model based on the identified hub gene was validated in two independent brain tissue datasets (GSE132903 and GSE5281), demonstrating favorable predictive performance. Considering the central role of neuroinflammation in AD, we further investigated immune cell infiltration profiles and their correlation with the hub gene. Potential small-molecule modulators targeting this gene were subsequently identified via DrugBank screening. This integrative approach provides novel insights into zinc-related molecular signatures in AD and their potential diagnostic and therapeutic applications.

## 2. Materials and Methods

### 2.1. Data Acquisition

**Figure 1:**
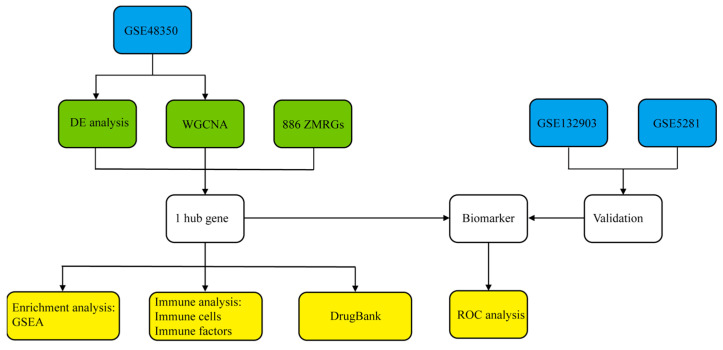
Brief workflow diagram. Blue represents the datasets used for analysis and validation, green represents the hub gene analysis methodology, and yellow represents the analytical methods used to validate the potential and function of hub genes as biomarkers.

For this investigation, we analyzed the gene expression dataset GSE48350, obtained from the Gene Expression Omnibus (GEO) repository. This dataset comprised a total of 253 brain tissue samples derived from four distinct regions (hippocampus, entorhinal cortex, superior frontal cortex, and post-central gyrus), which were categorized into two groups: 80 samples from patients with AD and 173 from non-demented (ND) control individuals. The GSE132903 dataset includes 195 middle temporal gyrus samples (98 ND and 97 AD). The GSE5281 dataset contains a total of 161 tissue samples. These samples were sourced from six distinct anatomical brain regions: the entorhinal cortex, hippocampus, medial temporal gyrus, posterior cingulate, superior frontal gyrus, and primary visual cortex (87 ND and 74 AD). All the ND samples were derived from healthy individuals. For the GSE48350 and GSE5281 datasets, AD diagnosis was determined using Braak staging and CERAD scores. For the GSE132903 dataset, cognitive levels were assessed by the MMSE, Global Deterioration Scale, and Montreal Cognitive Assessment. Demographic characteristic for AD and ND controls included in the datasets is provided in Supplementary Table S1. A list of 886 zinc metabolism-related genes (ZMRGs) was identified in a previously published study [20], which was derived by retrieving multiple zinc metabolism-related gene sets (specifically, those derived from Gene Ontology (GO) terms, REACTOME pathways, and Human Phenotype Ontology (HP) terms) from the MSigDB database. The methodological approach employed in this study is schematically illustrated in Figure 1.

### 2.2. Differential Expression Analysis

Differentially expressed genes (DEGs) in the AD cohort, relative to the ND cohort, were identified via the limma R package (v3.40.6). The analysis involved first fitting a multiple linear regression model to the gene expression data with the lmFit function. Subsequently, the eBayes function was used to moderate the standard errors via an empirical Bayes method, which generated moderated t-statistics and log-odds of differential expression. A gene was defined as a DEG if it met the significance thresholds of an adjusted *p*-value < 0.05 and an absolute log_2_ fold change (|log_2_FC|) > 0.585.

### 2.3. Weighted Gene Co-Expression Network Analysis (WGCNA)

The WGCNA (version 1.70.3) R package was employed to generate a weighted gene co-expression network from the stringently pre-processed data. The filtering stage involved the removal of 50% of genes with the lowest median absolute deviation (MAD) and the exclusion of outlier samples and genes via the goodSamplesGenes function. A scale-free network topology was achieved by transforming the pairwise Pearson’s correlation matrix into an adjacency matrix using a soft-thresholding power of β = 5. Specifically, for the soft thresholding power (β), we followed the recommendations of the WGCNA package. We selected the smallest β value that ensured the network approximated a scale-free topology (typically requiring an R^2^ of 0.8 or higher) while simultaneously maintaining a relatively low mean connectivity. This approach facilitates the construction of a robust and biologically more meaningful gene co-expression network. To identify gene modules, this adjacency matrix was converted to a Topological Overlap Matrix (TOM), and hierarchical clustering was applied to the resulting dissimilarity matrix (1 − TOM). We set a minimum module size of 30 genes and merged modules with an eigengene dissimilarity of less than 0.25. Ultimately, this analysis identified 25 co-expression modules, while genes not belonging to any module were assigned to the “grey” module.

### 2.4. Identification of Hub Genes

We identified candidate genes central to both AD pathogenesis and zinc metabolism by intersecting the DEGs, genes from the key WGCNA module, and a zinc metabolism gene set using the VennDiagram (version 1.7.3) R package. The differential expression of these intersecting genes was then visualized via violin plots. Statistical significance between the AD and ND groups was determined using the t-test for normally distributed data and the Mann–Whitney U test for non-normally distributed data, with a significance threshold set at *p* < 0.05.

### 2.5. Gene Set Enrichment Analysis (GSEA)

To elucidate the underlying biological functions, Gene Set Enrichment Analysis (GSEA v3.0) was performed. Samples were dichotomized into high- and low-expression cohorts based on the median expression value of *SLC30A3*. Using the KEGG gene sets from MSigDB (c2.cp.kegg.v7.4), enrichment scores were calculated based on 1000 permutations. Gene sets containing between 5 and 5000 genes were included, and those with a nominal *p* < 0.05 and an FDR < 0.25 were deemed significantly enriched.

### 2.6. Receiver Operating Characteristic (ROC) Analysis

The diagnostic performance of *SLC30A3* expression was assessed via ROC curve analysis using the pROC R package (v1.17.0.1). We quantified its diagnostic accuracy by calculating the area under the curve (AUC) and its corresponding 95% confidence interval, utilizing the package’s built-in ci function.

### 2.7. Immune Cell Infiltration and Immune-Related Factor Analysis

We characterized the immune context of each sample in the GSE48350 dataset by applying the CIBERSORT algorithm via the IOBR (version v0.99.9) R package to estimate the fractions of 22 immune cell types. To further explore the role of *SLC30A3* in immunomodulation, we performed Spearman’s correlation analysis between its expression and a panel of immune-related genes obtained from the TISIDB database. This panel comprised 18 immunosuppressive genes, 36 immunostimulatory genes, and 34 chemokines.

### 2.8. Drug Prediction from DrugBank

DrugBank (https://go.drugbank.com/) is a comprehensive drug database containing more than 7800 entries, including FDA-approved drugs, experimental compounds, and nutraceuticals [21]. To explore *SLC30A3* as a potential druggable target, we interrogated the DrugBank database to identify existing drugs that may modulate its activity.

### 2.9. Western Blot

SH-SY5Y cells (CL-0208, Procell, Wuhan, China) were cultured in DMEM/F12 medium (PM150312, Procell) supplemented with 10% fetal bovine serum (FBS, SH30084.03, Hyclone Logan, UT, USA) under 5% CO_2_ at 37 °C. Cells in the logarithmic growth phase were used for experiments and divided into two groups. For the AD model group, cells were treated for 24 h with culture medium containing 30 nmol/L okadaic acid (OKA, HY-N6785, MCE, Shanghai, China) to induce AD-like pathology. For the control group, the culture medium was replaced with fresh medium without OKA and incubated for the same duration. Cells from both groups were harvested, and total protein was extracted using RIPA lysis buffer (P0013B, Beyotime, Shanghai, China) containing 10% phenylmethylsulfonyl fluoride (PMSF, ST506, Beyotime). Protein concentrations were determined using a BCA protein assay kit (P0010, Beyotime), followed by denaturation through heating. Proteins were separated via SDS–PAGE and transferred onto polyvinylidene fluoride (PVDF) membranes (IPVH00010, Millipore, Boston, MA, USA). The membranes were blocked with 5% milk in TBST at room temperature for 1 h, and subsequently incubated overnight at 4 °C with the following primary antibodies: anti-SLC30A3 (17363-1-AP, Proteintech, Wuhan, China; 1:3000 dilution) and anti-GAPDH (60004-1-Ig, Proteintech; 1:5000 dilution). After washing, the membranes were incubated at room temperature for 1 h with corresponding secondary antibodies: anti-SLC30A3 (111-035-003, Jackson, Lancaster, PA, USA; 1:10,000 dilution) and anti-GAPDH (115-035-003, Jackson; 1:10,000 dilution). Immunoreactive bands were visualized using an ECL detection reagent (NCI5079, Thermo, Waltham, Massachusetts, USA), and band intensities were quantified by densitometric analysis using ImageJ software (version 1.53k).

## 3. Results

### 3.1. SLC30A3 Was the Hub Gene Associated with Zinc Metabolism and AD

**Figure 2:**
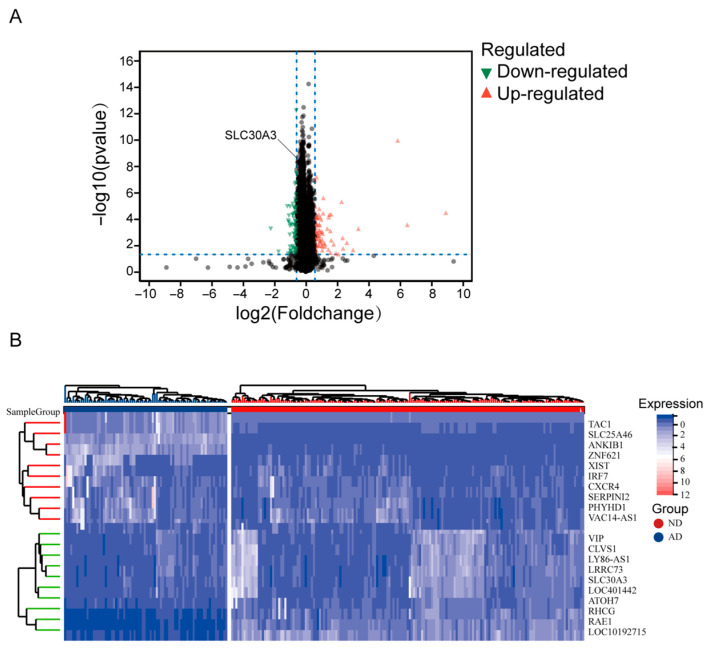
DEGs between AD and ND samples. (**A**) visualizes the overall results, with red and green dots representing genes with significantly higher expression in AD and ND, respectively (display range limited to |log2FC| ≤ 10). To highlight the most critical changes, a heatmap (**B**) was generated to visualize the expression patterns of the top 20 most DEGs between the disease and control cohorts.

Differential expression analysis was performed on the GSE48350 dataset to identify potential hub genes, with significance defined by the criteria of the |log_2_FC| > 0.585 and an adjusted *p*-value < 0.05. A comparison of gene expression between AD and ND samples revealed 160 DEGs, comprising 86 upregulated and 74 downregulated genes (Supplementary Table S2). To visualize these findings, we generated a volcano plot depicting the DEGs (Figure 2A) and a heatmap illustrating the expression of the 20 most significantly altered genes (10 upregulated and 10 downregulated) across samples (Figure 2B).

**Figure 3:**
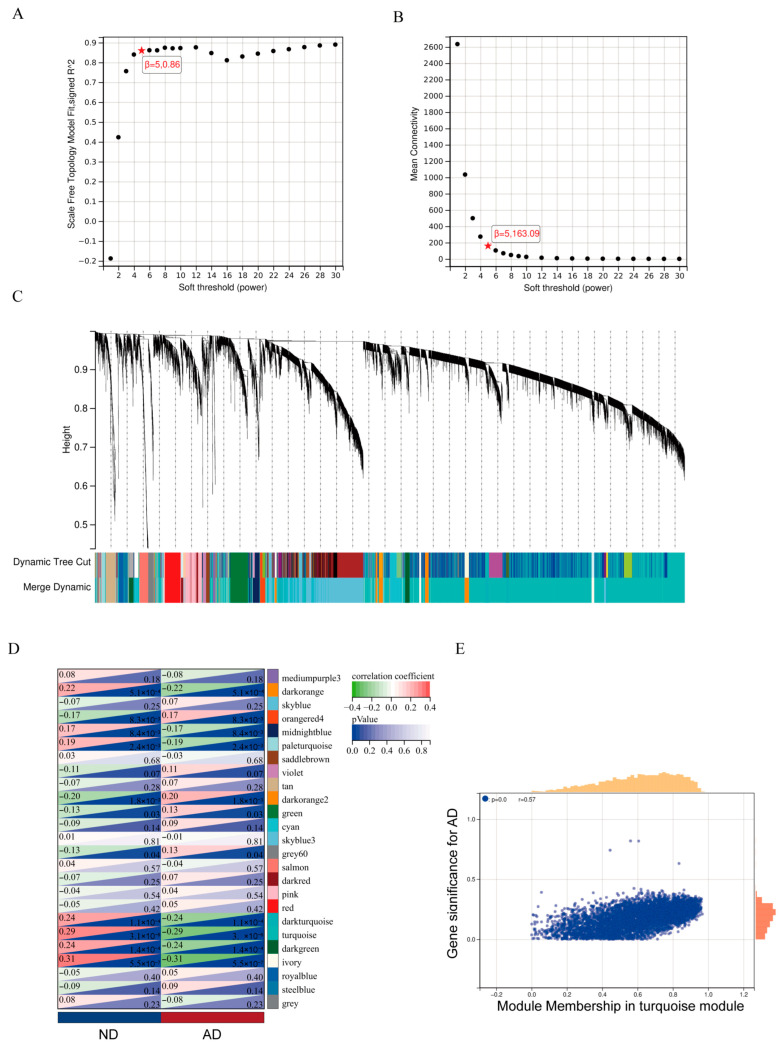
The WGCNA results of GSE48350. (**A**) Scale-free fit index (y-axis) versus the soft-thresholding power (β) (x-axis). (**B**) Mean connectivity (y-axis) versus the soft-thresholding power (β) (x-axis). (**C**) Hierarchical clustering dendrogram of genes, with identified co-expression modules assigned colors in the track below. (**D**) Heatmap showing the correlation between module eigengenes (rows) and clinical traits (columns). Each cell displays the correlation coefficient and the corresponding *p*-value. (**E**) Scatterplot of gene significance for a key clinical trait versus module membership for all genes within the turquoise module.

WGCNA was then conducted on the same dataset after removing outlier samples and aberrant data, resulting in 10,273 genes across 253 samples. A soft-thresholding power of 5 was selected for network construction, which resulted in a scale-free topology fit index of 0.86 while maintaining a mean connectivity of 163.09 (Figure 3A,B). Hierarchical clustering with dynamic tree cutting generated 25 distinct co-expression modules (merge threshold: 0.25; minimum module size: 30; sensitivity: 3; Figure 3C). Among these, correlation analysis with clinical traits indicated that the ivory module (r = −0.31, *p* = 5.5 × 10^−7^) and turquoise module (r = −0.29, *p* = 3.1 × 10^−6^) were most strongly and negatively correlated with AD (Figure 3D). Therefore, these two modules were selected for subsequent hub gene screening. To evaluate the biological relevance of the identified modules, we analyzed the correlation between module membership (MM) and gene significance (GS). For the turquoise module, we found a strong and significant positive correlation between MM and GS for the disease phenotype (r = 0.57, *p* < 0.05; Figure 3E), indicating that its genes are highly associated with the disease. A full gene list of the turquoise module is provided in Supplementary Table S3.

**Figure 4:**
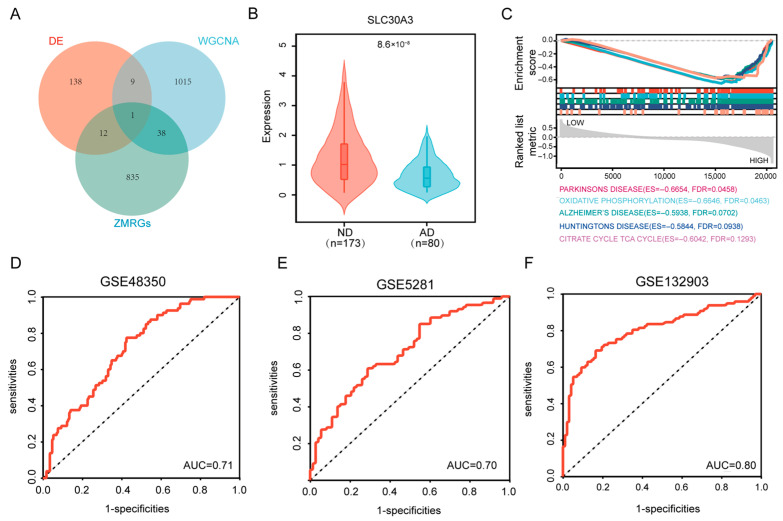
GSEA and hub gene analysis. (**A**) Venn diagram showing the intersection of three gene sets: DEGs between AD and ND, genes from the WGCNA turquoise module, and ZMRGs, identifying *SLC30A3* as the sole hub gene. (**B**) Violin plot comparing *SLC30A3* expression in ND and AD samples from the GSE48350 dataset. (**C**) GSEA results for pathways enriched with *SLC30A3* expression. (**D**–**F**) ROC curves assessing the diagnostic accuracy of *SLC30A3* for distinguishing AD from ND samples, with corresponding AUC values shown for three independent datasets: GSE48350 (**D**), GSE5281 (**E**), and GSE132903 (**F**).

By intersecting the 160 DEGs, 1063 turquoise module genes, and 886 ZMRGs, a single overlapping gene, *SLC30A3*, was identified, which was designated as the hub gene linking zinc metabolism and AD (Figure 4A). The violin plot demonstrated that *SLC30A3* expression was significantly reduced in AD compared with ND samples in GSE48350 (Figure 4B). Consistent downregulation was also observed in the independent datasets GSE132903 and GSE5281 (Supplementary Figure S1).

### 3.2. Pathways Enriched for SLC30A3

GSEA revealed that lower *SLC30A3* expression was significantly associated with several neurodegenerative disease pathways, including Parkinson’s disease (NES = −1.8708, FDR = 0.0458), AD (NES = −1.9613, FDR = 0.0702), Huntington’s disease (NES = −1.8832, FDR = 0.0938), and amyotrophic lateral sclerosis (ALS) (NES = −1.7313, FDR = 0.0623). In addition, multiple energy metabolism-related pathways were enriched, such as the citrate cycle (TCA cycle) (NES = −1.5295, FDR = 0.1293) and oxidative phosphorylation (NES = −1.8554, FDR = 0.0463). Pathways related to synaptic plasticity and neuronal signaling were also implicated, including long-term potentiation (NES = −1.8779, FDR = 0.0512), long-term depression (NES = −1.7903, FDR = 0.0534), calcium signaling pathway (NES = −1.8822, FDR = 0.0644), and axon guidance (NES = −1.6783, FDR = 0.0665) (Figure 4C). A full list of enriched pathways is provided in Supplementary Table S4.

### 3.3. Validation of the Diagnostic Model

We next evaluated the potential diagnostic utility of *SLC30A3* expression for distinguishing AD from ND samples. The model’s performance was validated in the GSE48350 dataset. The ROC curve analysis confirmed its utility, showing an AUC of 0.71 (Figure 4D). Validation in two independent datasets resulted in AUC values of 0.70 (GSE5281) (Figure 4E) and 0.80 (GSE132903) (Figure 4F), indicating that *SLC30A3* has promising potential as a biomarker for AD diagnosis.

### 3.4. SLC30A3 Affects the Immune Microenvironment of AD

The intricate network of immune cells, extracellular matrix components, inflammatory mediators, and growth factors collectively forms the brain microenvironment, which plays a pivotal role in driving the pathogenesis of AD. It influences neuroinflammation, neuronal viability, and disease progression, thereby representing a valuable target for biomarker discovery and therapeutic intervention.

**Figure 5:**
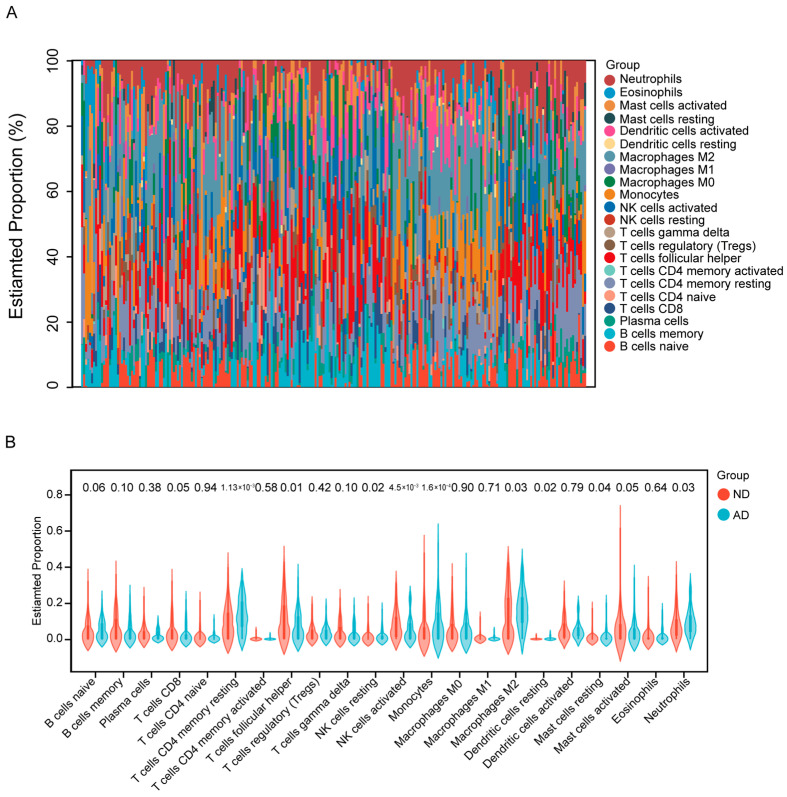
Immune cell infiltration analysis. (**A**) Heatmap illustrating the relative abundance of 22 immune cell types across each individual sample. (**B**) violin plot comparing the infiltration scores of each immune cell type between the AD and ND cohorts.

To characterize the immune cell landscape in AD, we analyzed 80 AD and 137 ND samples by computationally estimating the proportions of 22 immune cell types with the CIBERSORT algorithm (Figure 5A). Comparative analysis using violin plots (Figure 5B) revealed significant increases in resting CD4 memory T cells (*p* = 1.13 × 10^−3^), resting NK cells (*p* = 0.02), monocytes (*p* = 1.6 × 10^−4^), M2 macrophages (*p* = 0.03), resting dendritic cells (*p* = 0.02), resting mast cells (*p* = 0.04), and neutrophils (*p* = 0.03) in AD compared with ND. Conversely, follicular helper T cells (*p* = 0.01) and activated NK cells (*p* = 4.5 × 10^−3^) were significantly reduced in AD.

**Figure 6:**
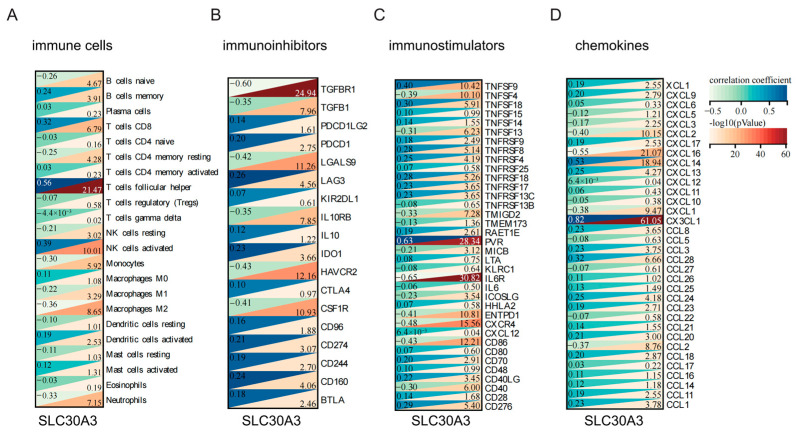
Correlation analysis between *SLC30A3* expression and immune-related markers. The plots show the correlation between *SLC30A3* mRNA levels and the infiltration scores or expression levels of: (**A**) various immune cell types, (**B**) a panel of immunoinhibitory molecules, (**C**) a panel of immunostimulatory molecules, and (**D**) a panel of chemokines. Correlation coefficients and *p*-values are displayed for each analysis.

Correlation heatmaps illustrated associations between *SLC30A3* expression and immune cell infiltration, immunoregulatory factors, immune stimulators, and chemokines. The correlation between *SLC30A3* expression and the infiltration levels of 22 distinct immune cell types was evaluated. The expression of *SLC30A3* showed significant positive correlations with six cell types: memory B cells, CD8 T cells, follicular helper T cells, activated NK cells, activated dendritic cells, and activated mast cells. In contrast, it was negatively correlated with naive B cells, resting CD4 memory T cells, resting NK cells, monocytes, M1 macrophages, M2 macrophages, and neutrophils. The strongest of these associations was the positive correlation with follicular helper T cells (Figure 6A).

In the immunosuppressive factor analysis, 15 out of 18 factors were significantly correlated with *SLC30A3* expression, with *TGFBR1* showing the highest significance but modest correlation strength, followed by *HAVCR2*, *LGALS9*, and *CSF1R* (Figure 6B). Among 36 immune stimulators, 26 were significantly associated, with *IL6R* and *PVR* showing the strongest correlations (Figure 6C). Of the 34 chemokines examined, 22 exhibited significant associations, with *CX3CL1* being the most strongly correlated (Figure 6D). Collectively, these findings suggest that *SLC30A3* may influence AD immunopathology by modulating both innate and adaptive immune cell subsets, as well as immune-related molecular mediators within the disease microenvironment.

### 3.5. Drugs Identified from DrugBank

A query of the DrugBank database identified two compounds targeting SLC30A3 (Table 1): zinc chloride and zinc sulfate. The term ‘unspecified form’ indicates that it does not specifically refer to a particular crystalline hydrate form (e.g., zinc sulfate heptahydrate, ZnSO_4_·7H_2_O) or its anhydrous form. Both zinc chloride and zinc sulfate are utilized as essential zinc supplements in total parenteral nutrition. Beyond this shared application, zinc chloride is also specifically used to treat established zinc deficiencies and their associated symptoms.

### 3.6. Downregulation of SLC30A3 Expression in an AD Cell Model

**Figure 7:**
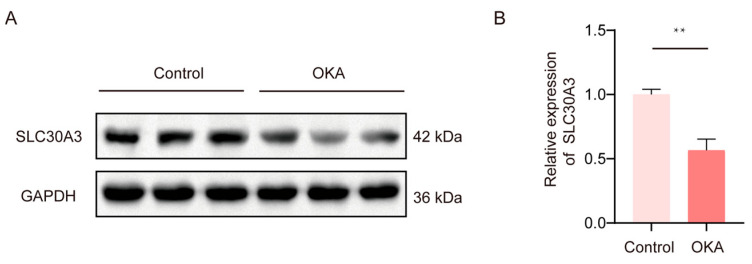
Relative expression analysis of SLC30A3 in the AD cell model. (**A**) Western blot analysis of SLC30A3 expression in the control and OKA-treated groups. GAPDH was used as an internal loading control for normalization. Three independent samples were loaded for each group. (**B**) Densitometric quantification of protein bands using ImageJ software to calculate the relative expression level of SLC30A3 in the AD model group. The data are presented as mean ± SD, ** representing *p* < 0.01.

To confirm that SLC30A3 expression is indeed dysregulated in Alzheimer’s disease, we established an AD cell model by treating SH-SY5Y cells with OKA. As shown in Figure 7, the expression level of SLC30A3 in the AD model was markedly reduced compared with the control group, exhibiting a statistically significant difference. Densitometric analysis revealed that its expression was only 56.6% of that observed in the control group, consistent with the results in Figure 4B (66.4%).

## 4. Discussion

AD is a multifactorial neurodegenerative disorder characterized by progressive cognitive decline, in which disturbances in metal ion homeostasis, especially zinc metabolism, have been increasingly recognized as important contributors to disease pathogenesis [13,22,23,24]. Accumulating evidence indicates that zinc dyshomeostasis can exacerbate hallmark AD pathologies, including Aβ deposition, tau hyperphosphorylation, oxidative stress, and synaptic dysfunction [14,19,25,26]. In the present study, we integrated differential expression analysis, WGCNA, and zinc metabolism–associated gene datasets to identify key zinc transport–related genes involved in AD. This approach revealed SLC30A3 as a zinc transporter downregulated in AD brain tissue, which exhibited robust predictive power (AUC = 0.70–0.80) across multiple cohorts, suggesting its potential as a novel diagnostic biomarker.

*SLC30A3* encodes ZnT3, which is predominantly localized in glutamatergic neurons and loads zinc into synaptic vesicles for activity-dependent release [27,28,29]. Synaptic zinc release modulates neurotransmission via NMDA and AMPA receptors and plays an essential role in long-term potentiation (LTP) [30,31,32]. Notably, ZnT3 knockout mice exhibit age-dependent memory impairment and synaptic loss [33,34,35]. Our GSEA supports these observations, showing that reduced *SLC30A3* correlates with enrichment in pathways linked to synaptic plasticity (LTP, LTD, calcium signaling, axon guidance), mitochondrial energy metabolism (TCA cycle, oxidative phosphorylation), and multiple neurodegenerative disorders (Parkinson’s disease, Huntington’s disease, ALS). These pathways are integral to neuronal survival and cognitive function, and their disruption is a recognized feature of early AD [36,37,38]. The energy metabolism associations in our data are consistent with prior reports showing that zinc deficiency impairs mitochondrial function, inhibits oxidative phosphorylation, and increases reactive oxygen species generation [39,40,41]. Impaired synaptic ZnT3-mediated zinc loading may thus compromise both mitochondrial homeostasis and activity-dependent synaptic signaling, two processes that are critically interconnected in the maintenance of cognitive function [42,43].

Accumulating evidence converges to suggest that zinc dyshomeostasis is not a peripheral phenomenon but a central, actionable element within the pathogenic framework of Alzheimer’s disease (AD), acting in concert with primary genetic and aging risks. The influence of zinc is multifaceted, directly exacerbating core AD pathologies. It promotes tau hyperphosphorylation and neurotoxicity [15], an effect that is therapeutically tractable through zinc chelation, leading to improved cognitive outcomes [44]. Zinc also plays a paradoxical role in amyloid-β (Aβ) dynamics: while its elevated concentration in plaques is a pathological feature [9], a finely tuned balance is required for Aβ’s own anti-amyloidogenic activities [45]. Furthermore, zinc is deeply intertwined with neuroinflammation, with studies demonstrating that its supplementation can therapeutically suppress inflammasome activation [46].

These molecular insights are mirrored in clinical findings, where AD patients consistently exhibit lower systemic zinc levels that correlate with inflammatory markers like CXCL10 [18]. The profound zinc enrichment in the hippocampus (up to 50 μg/g) [47], a region devastated by AD, highlights the brain’s delicate reliance on this metal. Cellularly, this balance is governed by a network of transporters (ZIP/ZnT) and metallothioneins [48,49], the failure of which can unleash cell death pathways including apoptosis, oxidative stress, and ferroptosis [50]. This positions zinc homeostasis as a critical node where diverse pathological insults converge. Consequently, strategies aimed at restoring this balance—whether by modulating transporters like ZnT3 [51] or through careful chelation [44,52]—are gaining significant traction as promising disease-modifying approaches. Thus, far from being a secondary effect, the regulation of zinc metabolism represents a fundamental process that actively shapes the course of AD.

One of the most striking findings in this study was the strong correlation between *SLC30A3* expression and immune infiltration patterns in the AD brain. We observed positive associations with memory B cells, CD8+ T cells, follicular helper T cells, activated NK cells, activated dendritic cells, and activated mast cells, as well as negative associations with resting CD4+ memory T cells, resting NK cells, monocytes, M1/M2 macrophages, and neutrophils. This suggests that SLC30A3 may influence both innate and adaptive immunity, potentially through microglia–neuron communication and modulation of cytokine/chemokine signaling, which warrants further investigation [53,54,55]. Notably, we observed a strong correlation with CX3CL1, a chemokine critical for microglial homeostasis and synapse preservation that is dysregulated in AD [56,57]. Zinc has been shown to modulate cytokine secretion and immune cell activation, and altered zinc transporter function could shift the balance toward a pro-inflammatory state [58,59]. Furthermore, compelling evidence indicates that zinc plays a pivotal role in modulating both innate and adaptive immune responses through a variety of molecular mechanisms. These include the regulation of enzyme function, intervention in signaling pathways, and the control of gene expression. Notably, the immunomodulatory effects of zinc are dose-dependent and context-specific to the underlying disease state [60,61,62]. By governing cellular zinc homeostasis, this raises the plausible hypothesis that SLC30A3 may influence immune regulation. Consequently, dysregulation of SLC30A3 could contribute to the onset and progression of Alzheimer’s disease by altering zinc-mediated immune functions.

The association of reduced *SLC30A3* expression with multiple neurodegenerative diseases in the GSEA results reinforces the importance of zinc in protein misfolding diseases [63,64]. It is plausible that zinc transporter deficits exacerbate protein aggregation and misfolding by altering metalloprotein activity, redox balance, and kinase/phosphatase signaling, thereby promoting tau phosphorylation and Aβ aggregation [11,45,65].

From a translational perspective, our DrugBank query identified Zinc chloride and Zinc sulfate as potential agents targeting SLC30A3. Zinc supplementation has been explored as a therapeutic strategy in AD, showing variable efficacy depending on formulation, dose, and patient zinc status [46,66]. However, because excessive zinc can be neurotoxic via promotion of Aβ aggregation and oxidative stress [67,68], targeted modulation of zinc transporters or neuron-specific delivery may be required to harness zinc’s neuroprotective effects while avoiding its deleterious consequences.

A key strength of our study is the use of transcriptomic analysis to identify a molecular signature linking zinc dyshomeostasis to AD pathology, reinforcing its role as a critical node and offering new research targets. However, this study has limitations. Our transcriptomic data are correlational and cannot establish causality. The analysis does not capture post-transcriptional or epigenetic changes, and potential confounders like comorbidities or medication may still have an influence. It is noteworthy that, although our findings in the AD cell model confirmed that SLC30A3 expression is indeed dysregulated and downregulated, further validation in animal models is required. In addition, it is essential to elucidate the potential mechanisms linking zinc homeostasis to AD and to explore the therapeutic potential of targeting zinc homeostasis, thereby bridging the gap between our experimental data and clinical application.

## 5. Conclusions

In conclusion, our study identifies *SLC30A3* as a novel AD-associated zinc transporter gene whose downregulation is linked to impaired synaptic plasticity, disrupted energy metabolism, and altered neuroimmune regulation. These results expand the current understanding of zinc biology in AD and suggest that modulation of SLC30A3 may represent a promising avenue for precision diagnostics and targeted therapeutics.

## Figures and Tables

**Table 1:** Drugs targeting the hub gene (*SLC30A3*) obtained from the DrugBank database.

Gene Symbol	Protein	UniProt ID	Name	Drug Group	Actions
*SLC30A3*	Zinc transporter 3 (ZNT3)	Q99726	Zinc chloride	approved	substrate
			Zinc sulfate, unspecified form	approved	substrate

## Data Availability

The data presented in this study are openly available in the article.

## Supplementary Materials

The following supporting information can be downloaded at https://www.mdpi.com/article/10.3390/genes16111380/s1. Table S1: Demographic characteristics for cases (AD) and non demented (ND) controls included in the datasets. Table S2: DEGs list in GSE48350 dataset; Table S3: Genes in the turquoise module; Table S4: List of enriched pathways in GSEA; Figure S1: Expression of *SLC30A3* in the GSE132903 and GSE5281 datasets.
