# Supplementary material for: SLC30A3 as a Zinc Transporter-Related Biomarker and Potential Therapeutic Target in Alzheimer’s Disease
Source: Genes (Basel). 2025 Nov 13;16(11):1380. doi: 10.3390/genes16111380 (PMC12651988; doi:10.3390/genes16111380)
Supplement: Supplementary file 1 [file genes-16-01380-s001.zip › Table S1-Demographic characteristics for cases (AD) and non demented (ND) controls included in the datasets.pdf]

Supplementary Table S1. Demographic characteristic for cases (AD) and non demented (ND) controls included in the datasets.

GSE48350

	ND Group (n=173)	AD Group (n=80)	*p*
**Age,y**			
mean	60.0	85.4	1.58E-26
Range	20-99	60-95	
**Gender,n**			
Men	91 (52.6%)	33 (41.3%)	
Women	82 (47.4%)	47 (58.7%)	

GSE132903

	ND Group (n=98)	AD Group (n=97)	*p*
**Age,y**			
mean	60.0	85.4	0.97
Range	70-102	70-98	
**Gender,n**			
Men	50 (51.0%)	49 (50.5%)	
Women	48 (49.0%)	48 (49.5%)	

GSE5281

	ND Group (n=74)	AD Group (n=87)	*p*
**Age,y**			
mean	78.4	79.8	0.40
Range	1-102	68-97	
**Gender,n**			
Men	53 (71.6%)	50 (57.5%)	
Women	21 (28.4%)	37 (42.5%)	
