# Supplementary figures and images for: SLC30A3 as a Zinc Transporter-Related Biomarker and Potential Therapeutic Target in Alzheimer’s Disease
Source: Genes (Basel). 2025 Nov 13;16(11):1380. doi: 10.3390/genes16111380 (PMC12651988; doi:10.3390/genes16111380)

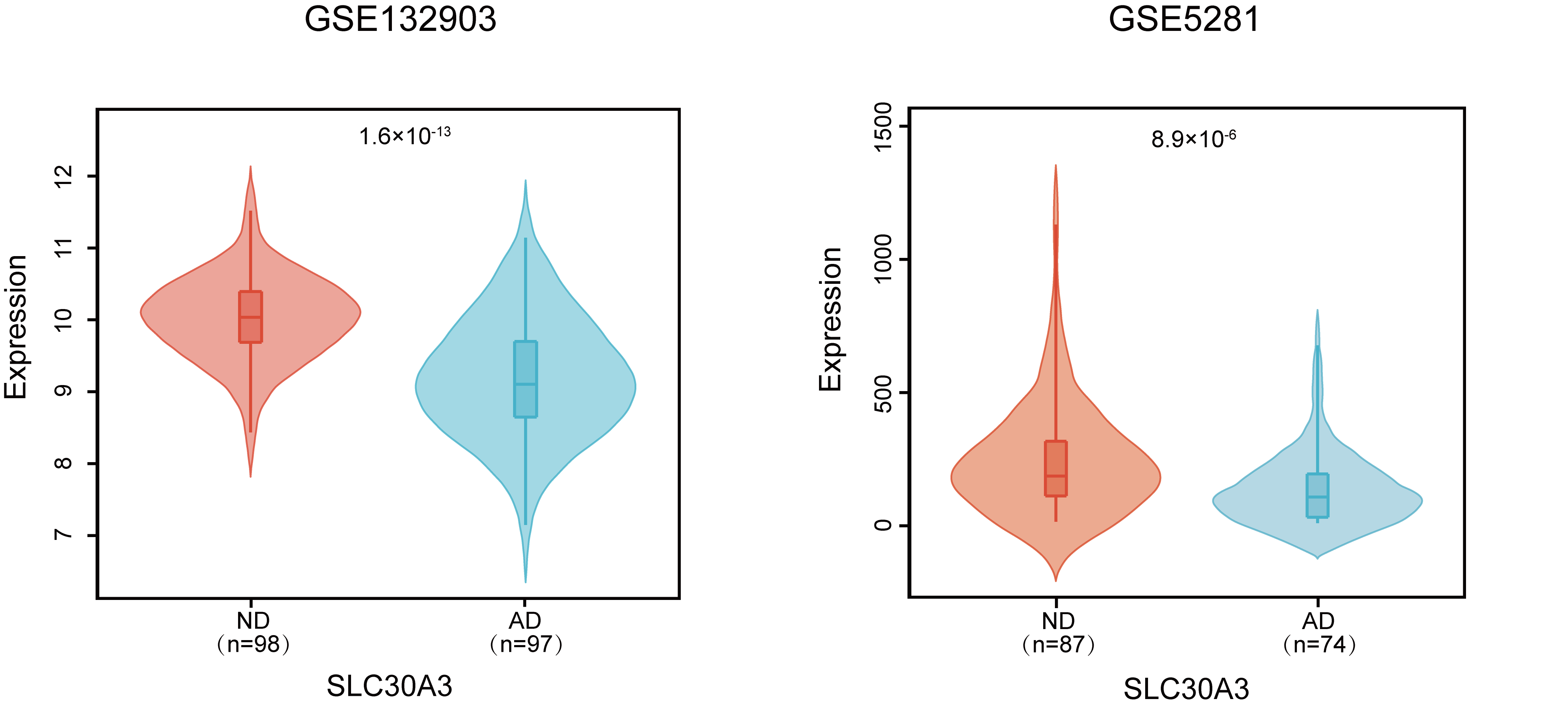

Supplement: Supplementary file 1 [file genes-16-01380-s001.zip › Figure S1-Expression of SLC30A3 in the GSE132903 and GSE5281 datasets.tif]
